# POPE: post optimization posterior evaluation of likelihood free models

**DOI:** 10.1186/s12859-015-0658-1

**Published:** 2015-08-20

**Authors:** Edward Meeds, Michael Chiang, Mary Lee, Olivier Cinquin, John Lowengrub, Max Welling

**Affiliations:** 1 Informatics Institute, University of Amsterdam, Amsterdam, The Netherlands; 2 School of Biological Sciences, University of California, Irvine, USA; 3 Department of Mathematics, University of California, Irvine, USA; 4 Donald Bren School of Informatics, University of California, Irvine, USA

**Keywords:** Approximate Bayesian computation, Simulation-based science, Bayesian inference

## Abstract

**Background:**

In many domains, scientists build complex simulators of natural phenomena that encode their hypotheses about the underlying processes. These simulators can be deterministic or stochastic, fast or slow, constrained or unconstrained, and so on. Optimizing the simulators with respect to a set of parameter values is common practice, resulting in a single parameter setting that minimizes an objective subject to constraints.

**Results:**

We propose *algorithms* for post optimization posterior evaluation (POPE) of simulators. The algorithms compute and visualize all simulations that can generate results of the same or better quality than the optimum, subject to constraints. These *optimization posteriors* are desirable for a number of reasons among which are easy interpretability, automatic parameter sensitivity and correlation analysis, and posterior predictive analysis. Our algorithms are simple extensions to an existing simulation-based inference framework called approximate Bayesian computation. POPE is applied two biological simulators: a fast and stochastic simulator of stem-cell cycling and a slow and deterministic simulator of tumor growth patterns.

**Conclusions:**

POPE allows the scientist to explore and understand the role that constraints, both on the input and the output, have on the optimization posterior. As a Bayesian inference procedure, POPE provides a rigorous framework for the analysis of the uncertainty of an optimal simulation parameter setting.

## Background

In science and industry alike, modelers express their expert knowledge by building a simulator of the phenomenon of interest. There is an enormous variety of such simulators, deterministic or stochastic, fast or slow, with or without constraints. For most simulators, e.g. driven by stochastic partial differential equations, it is impossible to write down an expression for the likelihood, which can make it highly challenging to optimize the simulator over its free parameters. This “blind optimization problem” is receiving increasing attention in the machine learning community [1–3].

However, even if the optimal parameter value ***θ*** ^⋆^ is found, this still leaves the scientist in the dark with respect to important questions such as: “Which parameters are correlated?”; “Which parameters are robust and which are sensitive?”; “Is my model overfitting, underfitting or just right”? We believe that methods capable of handling these type of questions post optimization are essential to the field of simulation-based modeling. In this paper we propose a new Bayesian framework that allows the scientist to answer these questions by defining a posterior distribution over all parameters that can be interpreted as “the probability that the outcome of a simulation conducted at that parameter value will result in a value of the objective that is equally good or better than a certain value $y^{\star }_{1}$, subject to certain constraints on both parameters as well as simulation outcomes”. This “Post Optimization Posterior Evaluation” (POPE) is different from standard ABC [4–6] in that standard ABC compares simulator outcomes with observations while POPE reasons about an optimization problem (subject to constraints). For instance, POPE can be meaningfully applied to an optimization problem without a single observation by asking which parameter values are expected to perform better than a certain threshold value on the objective. While different philosophically, POPE can be implemented by using one-sided kernels within an ABC framework.

POPE is not intended to be an optimization tool for likelihood-free models. While one can use the POPE framework to iteratively and adaptively optimize an objective, its core use is in quantifying and visualizing the full distribution over parameters, including their posterior interactions, that result in equally good or better objective values than some given $y^{\star }_{1}$. One could for instance imagine using Bayesian optimization [3] or some other global optimization technique [7] to find a value for $y^{\star }_{1}$ and then visualize the posterior distribution of parameters given that value. The posterior distribution we approximate using ABC sampling techniques is related to the concept of “probability of improvement” often used in Bayesian optimization [8] to measure how promising a parameter value is in terms of improving on the current best solution. However, in that context the probability of improvement includes, besides uncertainty due to the stochastic nature of simulation, also the uncertainty of a surrogate model’s ability to predict the value of *y*_1_. In contrast, with POPE the probability of improving the current best solution is only determined by the noise in the simulation.

POPE addresses the requirements of simulation-based science by providing tools that have a number of properties that are beneficial to a scientist: 1) the posterior distribution over parameters has a clear and interpretable meaning and can be used to suggest alternative parameters to explore, 2) POPE can handle multiple objectives and constraints, 3) unlike most standard optimization methods, POPE can handle simulators with stochastic outputs and complicated input or output constraints, 4) POPE can handle multimodal posterior distributions, 5) as part of its computation POPE will generate posterior predictive samples that can be used to evaluate the model fit, and 6) by incorporating Gaussian process surrogate models it can handle expensive simulators.

In this paper we will develop POPE and apply it to two real-world cases: one fast stochastic simulator in the domain of stem cell biology and one slow deterministic simulator developed for cancer research.

### Approximate Bayesian computation

One of the primary goals of Bayesian inference is to draw samples from the following (usually intractable) posterior distribution:

(1) $$ \pi(\boldsymbol{\theta} | \mathbf{y}^{\star}_{1}, \ldots, \mathbf{y}^{\star}_{N}) \propto \pi(\boldsymbol{\theta}) \pi(\mathbf{y}^{\star}_{1}, \ldots, \mathbf{y}^{\star}_{N} | \boldsymbol{\theta})  $$

where *π*(***θ***) is a prior distribution over parameters ***θ***∈IR^*D*^ and $\pi (\mathbf {y}^{\star }_{1}, \ldots, \mathbf {y}^{\star }_{N} | \boldsymbol {\theta })$ is the likelihood of *N* data observations, where $\mathbf {y}^{\star }_{n} \in \text {I\!R}^{J}$. The vector of *J* values can either be “raw” observations or, more typically, informative statistics of observations. In this paper we consider the case where *N*=1 (though all our methods apply equally to *N*>1) and will henceforth drop the subscripts. The unconventional superscript on **y** ^⋆^ is used to distinguish the observations from the simulator outputs **y**.

In ABC the likelihood function *π*(**y** ^⋆^|***θ***) is usually not available as a function but rather as a complex simulation, hence the alternative name for ABC, *likelihood-free inference*. ABC sampling algorithms treat the simulator as an auxiliary variable generator and discrepancies between the simulator outputs and the observations as proxies for the likelihood value. If we let $\mathbf {y}\, {\overset {\text {sim}}\sim }\, \pi (\mathbf {y} | \boldsymbol {\theta })$ be a “draw” from the simulator, the likelihood can be written as:

(2) $$ \pi\left(\mathbf{y}^{\star} | \boldsymbol{\theta}\right) = \int \left[\mathbf{y} = \mathbf{y}^{\star}\right] \pi(\mathbf{y} | \boldsymbol{\theta}) d\mathbf{y}   $$

where [·]=1 if the arguments are true, and 0 otherwise. Equation 2 implies that we can compute the exact likelihood by integrating over all possible simulation output values. In reality, since this integral requires simulations to match observations exactly, it is only achievable for discrete data. For continuous **y** ^⋆^, *J* slack variables ***ε*** are introduced around **y** ^⋆^. More specifically, an ***ε***-kernel function *π*_***ε***_ is used to measure the discrepancy between simulation results and observations. In practice the likelihood is approximated by a Monte Carlo estimate computed from *S* draws of the simulator $\mathbf {y}^{(s)} {\overset {\text {sim}}\sim } \pi (\mathbf {y} | \boldsymbol {\theta })$:

(3) $$ \pi_{\boldsymbol{\epsilon}}(\mathbf{y}^{\star} | \boldsymbol{\theta}) = \int \pi_{\boldsymbol{\epsilon}}(\mathbf{y}^{\star} | \mathbf{y}) \pi(\mathbf{y} | \boldsymbol{\theta}) d\mathbf{y} \approx \frac{1}{S} \sum_{s=1}^{S} \pi_{\boldsymbol{\epsilon}}\left(\mathbf{y}^{\star} | \mathbf{y}^{(s)}\right)   $$

Although Eq. 3 is an *unbiased* estimator of *π*_***ε***_(**y** ^⋆^|***θ***), this ABC likelihood is an approximation to the true likelihood, since *π*_***ε***_(**y** ^⋆^|***θ***)≈*π*(**y** ^⋆^|***θ***). In other words, ***ε*** puts the “approximate” in ABC; samples are drawn from the true posterior only as ***ε***→0. Common *π*_***ε***_ kernels are the ***ε***-tube $\pi _{\boldsymbol {\epsilon }}(\mathbf {y}^{\star } | \mathbf {y}) \propto \prod _{j}\left [ \| y_{j}^{\star }-y_{j} \|_{1} \leq \boldsymbol {\epsilon }_{j} \right ]$ and the Gaussian kernel $\pi _{\boldsymbol {\epsilon }}(\mathbf {y}^{\star } | \mathbf {y}) = \prod _{j}\mathcal {N} \left (y_{j}^{\star } | y_{j}, \boldsymbol {\epsilon }_{j}^{2} \right)$.

Among the many possible ABC sampling algorithms, Markov chain Monte Carlo (MCMC) ABC is of particular relevance to this work [4–6]. In the Metropolis-Hastings (MH) step the proposal distribution is composed of the product of the proposal for the parameters ***θ*** and the proposal for the simulator outputs:

(4) $$ q\left(\boldsymbol{\theta}^{\prime}, \mathbf{y}^{\prime} | \boldsymbol{\theta}\right) = q\left(\boldsymbol{\theta}^{\prime} | \boldsymbol{\theta}\right) \pi\left(\mathbf{y}^{\prime} | \boldsymbol{\theta}^{\prime}\right)  $$

i.e. parameters ***θ*** ^′^ are first proposed, then outputs **y** ^′^ are generated from the simulator with input parameters ***θ*** ^′^.

Using this form of the proposal distribution, and using the Monte Carlo approximation Eq. 3, we arrive at the following Metropolis-Hastings accept-reject probability,

(5) $$ \alpha = \min \left(1, \frac{\pi\left(\boldsymbol{\theta}^{\prime}\right) \sum_{s=1}^{S} \pi_{\boldsymbol{\epsilon}}(\mathbf{y}^{\star} | \mathbf{y}^{\prime(s)}) q(\boldsymbol{\theta} | \boldsymbol{\theta}^{\prime})}{\pi\left(\boldsymbol{\theta}\right) \sum_{s=1}^{S} \pi_{\boldsymbol{\epsilon}}(\mathbf{y}^{\star} | \mathbf{y}^{(s)}) q(\boldsymbol{\theta}^{\prime} | \boldsymbol{\theta})} \right)   $$

When only the numerator is re-estimated at every iteration (and the denominator is carried over from the previous iteration), then this algorithm corresponds to pseudo-marginal (PM) sampling [9, 10]. PM sampling is asymptotically correct (taking for granted the approximation introduced by the kernel *π*_***ε***_) but can display very poor mixing properties. By resampling the denominator as well, we improve mixing at the cost of introducing a further approximation. This sampler is known as the marginal sampler [4, 6]. There is evidence that using a single simulation is adequate [11]; indeed, we set *S*=1 in our experiments and found no benefit to tuning *S*.

For expensive simulators, even a single simulation per MH step can make ABC-MCMC infeasible.

Surrogate modeling—where the history of all simulations are stored in memory and used to build a surrogate of the simulator—may be the only option to make progress in that case.

## Methods

In regular ABC the simulator generates output statistics **y** that are compared directly with observations **y** ^⋆^. For optimization problems, however, the scientist may interpret *y*_1_ as a cost and $y^{\star }_{1}$ as an estimate of the minimum cost. Other simulation statistics {*y*_*j*_},*j*=2..*J* may be constrained, e.g. $\{y_{j} \leq y^{\star }_{j} \}.$ For instance, the cost could be some measure of misfit between simulator outcomes and desirable outcomes while constraints could represent domains within which certain simulation results should lie (constraints can of course also be incorporated into the cost function, but as we will see, it is sometimes beneficial to treat them separately). Our first guess to elucidate some posterior distribution over parameters could be to define a Gibbs distribution *p*(*y*_1_)∝ exp(−*β* *y*_1_) which we would treat as a likelihood similar to *π*_***ε***_ and apply ABC, rejecting everything that does not satisfy the constraints. Unfortunately, we do not consider this a satisfactory solution because the posterior does not have a clear interpretation. For instance, simply scaling the arbitrary constant *β* would change the posterior.

A better solution is to define a new type of (one-sided) Heavyside kernel in ABC: $\left [ y_{1} \leq y^{\star }_{1} \right ]$ which is 1 when the argument is satisfied and 0 otherwise. Note that this kernel is applied to both the objective *y*_1_ and the constraints {*y*_*j*_} alike. The quantity $y^{\star }_{1}$ is given by the lowest value of the objective found by some optimization procedure (e.g. grid-search, black-box [7] or Bayesian optimization [3], etc). The posterior samples produced by an ABC algorithm that uses this one-sided kernel have a very clean interpretation, namely they represent *the probability that a simulation run at that parameter value will generate an equally good or better value for the objective while satisfying all the constraints*. This distribution can be used to suggest new regions to explore (e.g. other modes, or regions that are farther away from constraint surfaces), and to visualize dependencies between parameters and their sensitivities.

The posterior described above thus corresponds to

(6) $$ \begin{aligned} &\pi(\boldsymbol{\theta} | \mathbf{y}^{\star}) \propto \pi(\boldsymbol{\theta}) \int \left[ \mathbf{y} \leq \mathbf{y}^{\star} \right] \pi(\mathbf{y} | \boldsymbol{\theta}) d\mathbf{y} \propto \pi(\boldsymbol{\theta})\\ &\quad \times \int_{-\infty}^{\mathbf{y}^{\star}} \pi(\mathbf{y} | \boldsymbol{\theta}) d\mathbf{y} \propto \pi(\boldsymbol{\theta}) F_{\mathbf{y} | \boldsymbol{\theta}}(\mathbf{y}^{\star}) \end{aligned}  $$

where *F*_**y**|***θ***_ is the cumulative distribution function (CDF) of the conditional probability density function *π*(**y**|***θ***) (or the probability of satisfying the constraint or improving the objective^1^). Since in ABC we cannot compute the likelihood analytically, it is approximated by a Monte Carlo estimate:

(7) $$ F_{\mathbf{y} | \boldsymbol{\theta}}(\mathbf{y}^{\star}) \approx \frac{1}{S} \sum_{s=1}^{S} \left[ \mathbf{y}^{(s)} \leq \mathbf{y}^{\star} \right] ~~~~~~~~~~~~~~~~~~~~~~ \mathbf{y}^{(s)} {\overset{\text{sim}}\sim} \pi(\mathbf{y} | \boldsymbol{\theta})  $$

Using the one-sided kernel [**y**≤**y** ^⋆^] will cause the ABC sampler to get stuck when initialized in a region where **y**>**y** ^⋆^ because every proposed sample will get rejected. Even when initialized in a region where **y**≤**y** ^⋆^, this kernel will make it very difficult to move between different “islands” (modes) in parameter space where these conditions hold. This problem is aggravated in high dimensions where $\left [ \mathbf {y} \leq \mathbf {y}^{\star } \right ] = \prod _{j} \left [ y_{j} \leq y_{j}^{\star } \right ] $ and every condition needs to be satisfied for the likelihood to be non-zero. A one-sided ***ε***-tube [**y**≤**y** ^⋆^+***ε***] adds some relief but suffers the same problem for most useful values of ***ε***.

The solution to this problem is to soften the kernel analogously to the softening of the condition [**y**=**y** ^⋆^] into *π*_***ε***_(**y** ^⋆^|**y**) in generalized ABC [5]. By using a soft kernel, the goodness of two sets of statistics can be computed and compared. If we define $d_{j} = y_{j} - y_{j}^{\star }$, then these soft kernels treat all simulation outputs less than $y_{j}^{\star }$ with likelihood proportional to 1 and provide quadratic or linear penalties otherwise. For example, a one-sided Gaussian kernel for the *j* statistic (or output constraint) is defined as

(8) $$\begin{array}{*{20}l} K_{\boldsymbol{\epsilon}_{j}}\left(y_{j};\; y_{j}^{\star} \right) = \left[ d_{j} \geq 0 \right] + \left[ d_{j} < 0 \right] \exp\left(-\frac{1}{2} \left(\frac{d_{j}}{\boldsymbol{\epsilon}_{j}}\right)^{2}\right) \end{array} $$

and a one-sided exponential kernel (i.e. linear penalty) is defined as

(9) $$\begin{array}{*{20}l} K_{\boldsymbol{\epsilon}_{j}}\left(y_{j};\; y_{j}^{\star} \right) = \left[ d_{j} \geq 0 \right] + \left[ d_{j} < 0 \right] \exp\left(-\frac{d_{j}}{\boldsymbol{\epsilon}_{j}} \right) \end{array} $$

By modifying ***ε*** we can control the relative importance or severity of the penalty, allowing us to use annealing schedules that adapt ***ε*** during the MCMC run in order to focus the sampling at modes when ***ε*** is small.

Up to this point we have only discussed *one-sided* likelihoods, but there is nothing preventing the likelihoods to incorporate both upper and lower constraints:

(10) $$ \pi\left(\mathbf{y}^{\star} | \boldsymbol{\theta} \right) = \int_{\mathbf{y}^{\star}_{a}}^{\mathbf{y}^{\star}_{b}} \pi(\mathbf{y} | \boldsymbol{\theta}) d\mathbf{y} = F_{\mathbf{y} | \boldsymbol{\theta}}(\mathbf{y}^{\star}_{b})-F_{\mathbf{y} | \boldsymbol{\theta}}(\mathbf{y}^{\star}_{a})  $$

The one-sided kernels are easily modified for this, setting the likelihood to 1 in between the regions, with quadratic or linear penalties outside of the regions.

### Modeling the simulator response

We may want to consider modeling the simulator response *π*(**y**|***θ***) if the outcome of the simulator is stochastic or the simulator is expensive to run. In the first case, we can reduce the variance of the Markov chain by learning a *local response model* ^2^ for every state ***θ***. For the second case, a *global response model* (a.k.a. a surrogate) over the entire ***θ***-space is more appropriate because it stores and makes use of the entire simulation history to predict responses at new ***θ*** locations.

#### Local response models

When the simulator is fast and stochastic, it can be beneficial to the inference procedure to build a local, conditional model of the distribution *π*(**y**|***θ***) using *S* simulator responses in $\mathbf {y}^{(1)}, \ldots, \mathbf {y}^{(S)} {\overset {\text {sim}}\sim } \pi (\mathbf {y} | \boldsymbol {\theta })$. The simplest local response model is the *conditional Gaussian*, an approach called *synthetic likelihood* in ABC [12]. It computes estimators of the first and second moments of the responses and uses the Gaussian distribution to analytically compute the likelihood (thus providing an alternative to kernel ABC). For our algorithms, this allows the direct computation of the CDF:

(11) $${} {\fontsize{9.2pt}{9.6pt}\selectfont{\begin{aligned} &\hat{\boldsymbol{\mu}}_{{\boldsymbol{\theta}}} = \frac{1}{S} \sum_{s=1}^{S} \mathbf{y}_{s} ~~~~~~~~~ \hat{\boldsymbol{\Sigma}}_{\boldsymbol{\theta}} = \frac{1}{S-1} \sum_{s=1}^{S} \left(\mathbf{y}^{(s)} - \hat{\boldsymbol{\mu}}_{{\boldsymbol{\theta}}} \right)\left(\mathbf{y}^{(s)} - \hat{\boldsymbol{\mu}}_{{\boldsymbol{\theta}}} \right)^{T} \end{aligned}}}  $$

(12) $$\begin{array}{@{}rcl@{}}{} F_{\mathbf{y} | \boldsymbol{\theta}}\left(\mathbf{y}^{\star}; \hat{\boldsymbol{\mu}}_{{\boldsymbol{\theta}}}, \hat{\boldsymbol{\Sigma}}_{\boldsymbol{\theta}}\right) = \int_{-\infty}^{\mathbf{y}^{\star}} \mathcal{N}\left(\mathbf{y} | \hat{\boldsymbol{\mu}}_{{\boldsymbol{\theta}}}, \hat{\boldsymbol{\Sigma}}_{\boldsymbol{\theta}} \right) d \mathbf{y} \end{array} $$

where $\hat {\boldsymbol {\mu }}_{{\boldsymbol {\theta }}}$ and $\hat {\boldsymbol {\Sigma }}_{\boldsymbol {\theta }}$ are computed from the *S* simulations. In experiments we can limit the number of parameters by using a factorized model: $\mathcal {N}\left (\mathbf {y} | \hat {\boldsymbol {\mu }}_{{\boldsymbol {\theta }}}, \hat {\boldsymbol {\Sigma }}_{\boldsymbol {\theta }}\right) \approx \prod _{j=1}^{J} \mathcal {N}\left (y_{j} | \hat {\mu }_{j}, \hat {\sigma }^{2}_{j}\right)$, resulting in a factorized product over CDFs as well. Modeling the response by only the first two moments may be inadequate due to multi-modality, asymmetric noise, etc. For such cases a *conditional KDE* (kernel density estimate) response model can by used. In [13] this approach is shown to be superior to conditional Gaussians for certain computational psychology models. Note that for Gaussian kernels the conditional KDE is very similar to kernel ABC, but has additional flexibility of adaptively choosing bandwidths (rather than the fixed ***ε*** in kernel ABC).

#### Global response models

For very expensive simulators it is impractical to run simulations at each parameter location in the MCMC run. In these cases it is worth the extra storage and the computational overhead of learning a model of the simulator response surface or surrogate. For global response models the Metropolis-Hastings diverges from ABC-MCMC in that simulations are only performed if the surrogate is very uncertain. When the surrogate is confident, no simulations are performed.

The natural global extension of the Gaussian conditional model is the Gaussian process (GP). The GP has been used extensively for surrogate modeling [1, 2, 8, 14, 15], including more recent applications in accelerating ABC [16, 17]. In [16] GPs directly model the log-likelihood in successive waves of inference, each one eliminating regions of low posterior probability. This approach is capable of handling high-dimensional simulator outputs. In [17] each dimension of the simulator response is modeled by a GP and explicitly uses the surrogate uncertainty to determine simulation locations (design points). The advantage of this approach is that CDFs can be computed directly from the GPs predictive distributions. A global extension of the conditional KDE is more complicated, but estimators such as the Nadayara-Watson could provide the necessary modeling machinery. These extensions are beyond the scope of this paper.

### MCMC for POPE

Algorithm 1 provides the pseudo-code for running a kernel ABC-MCMC version of POPE (easily modified to accommodate response models by plugging in the appropriate likelihood function for *π*_***ε***_(**y** ^⋆^|**y** ^(*s*)^)). This is simply ABC-MCMC with one-sided kernel likelihoods. There are two possible modes for running POPE: marginal and pseudo-marginal. When running marginal MCMC, the state of the Markov chain only includes ***θ***, and, as discussed earlier, has the property of improved mixing with the cost of doubling the number of simulations per Metropolis-Hastings step and a less accurate posterior. On the other hand, pseudo-marginal can mix poorly, but uses fewer simulations and is more accurate. Choosing between the two modes is problem specific.



#### Adaptive POPE

In ABC, the choice of ***ε*** is crucial to both the MCMC mixing and the precision of the posterior distribution. There is an obvious trade-off between the two as large ***ε*** provides better mixing but poorer approximations to the target distribution. It is common in ABC to adapt ***ε*** using quantiles of the discrepancies (e.g. in Sequential Monte Carlo ABC [18]) or using a more complicated approach, for example based on the threshold acceptance curve [19], or to include ***ε*** as part of the state of the Markov chain [20].

We propose an online version of the quantile method (see function *UpdateEpsilons* in Algorithm 2), setting ***ε*** to a quantile of the exponential moving average (EMA) of the discrepancies or some minimum values ***ε*** ^MIN^, which ever is greater. Minimum values ***ε*** ^MIN^ are set not only for computational reasons, but also to reflect the scientist’s intuition regarding the relative importance of the constraints. Because ***ε*** can fluctuate during the MCMC run, it can explore regions where some constraints are easily satisfied, but others are not, and vice-versa. A quantile parameter *β* puts pressure on the chain to keep ***ε*** small.



For some problems we may not know certain *objective values* in **y** ^⋆^ before running POPE. For these cases simple adaptive MCMC procedures can estimate **y** ^⋆^ during the MCMC run. For deterministic simulators, **y** ^⋆^ can be updated after each simulation. For stochastic simulators we propose a local averaging procedure based on the EMA of **y**, similar to the adaptation of ***ε***. The intuition behind this is that the best objective value **y** ^⋆^ at ***θ*** ^⋆^ is the expected value of the simulator response at ***θ*** ^⋆^. An EMA of the simulation response approximates this expectation and we have found in our experiments with stochastic simulators that it performs well and conveniently fits into the POPE MCMC procedure (i.e. there is no need to set up an entirely different optimization procedure with complicated constraints on the input and outputs since these are already part of POPE). This is function *UpdateObjectives* in Algorithm 2.

These are adaptive MCMC algorithms that do not necessarily target the correct posterior distribution. The simplest way to correct this is to simply use a few MCMC runs to set ***ε*** or **y** ^⋆^ (if needed) or stop the adaptation altogether after a burnin period, from that point using non-adaptive ABC-MCMC. This is the approach we took in our experiments. Alternatively, the adaptation decay rate parameters *δ* and *γ* in Algorithm 2, could slowly increase to 1, at which point the adaptation ceases.

#### Posterior analysis of MCMC results

Along with the posterior parameter distribution *p*(***θ*** | **y** ^⋆^), which is usually the main distribution of interest in a Bayesian analysis, we will also examine the *posterior predictive distribution*, denoted as *p*(**y**|**y** ^⋆^), though perhaps unintuitive, is the distribution of statistics (the predictions) generated by the simulation at the parameters from *p*(***θ*** | **y** ^⋆^). Posterior predictive distributions are used in statistics for *model checking* and *model improvement* [21], for example, and use the generative model with parameters from the posterior to generate pseudo or replicated data. Statistics of this data, defined by the statistician and considered important for the problem at hand, are compared to the statistics from the observations (the real data). One can then examine the bias and variance of the posterior predictive distributions with respect to the observations **y** ^⋆^, or perform Bayesian t-tests (how probable are the observations **y** ^⋆^ under *p*(**y**|**y** ^⋆^)) (see [21], Chapter 6).

For ABC, the posterior analysis comes naturally, and, usually, for free. Using ABC-MCMC algorithms, statistics (judged important a priori by the scientist) are generated at each Metropolis-Hastings step. Simply storing the pairs {**y**,***θ***} from the MH step is sufficient to produce both *p*(**y** | **y** ^⋆^) and *p*(***θ*** | **y** ^⋆^). In addition to the posterior predictive, visualizing the input-output posteriors, i.e. a joint *p*(*y*_*j*_,*θ*_*d*_ | **y** ^⋆^) from the combined posterior predictive and posterior distribution, can lead to additional insight.

## Results and discussion

### Case 1: stem-cell niche geometry in C. elegans

Minimizing the time it takes to develop an organ or to return to a desired steady state after perturbation is an important performance objective for biological systems [22, 23]. Control of the cycling speed of stem cells and of the timing of their differentiation is critical to optimize the dynamics of development and regeneration. This control is often exerted in part by stem cell niches. While stem cell niches are known to employ a number of molecular signals to communicate with stem cells [24], the impact of their geometry on stem cell behavior has received less attention. To begin to address this question, we ask here how niches should be shaped to minimize the amount of time to produce a given number of differentiated cells.

We consider a model organ inspired from the C. *elegans* germ line, which is similar to a number of other systems [25]. Cells reside within a tube-like structure; one end defined by the niche is closed, while the other is open and allows cells to exit. The set of possible positions that can be assumed by stem cells is constrained by the geometry of the niche; a dividing cell that is surrounded by neighbors pushes away one of its neighbors, which in turn might need to push away one of its own neighbors; cells pushed outside of the niche by one of these chain displacement reactions are forced to leave the cell cycle and differentiate. A simulator we developed tracks cell division and movement, and outputs the time it takes to produce N cells for a given geometry. This geometry is such that rows are defined along the main axis of the organ; each cell row has its own size, comprised between 1 and 400 cells. There are several constraints that are put on the niche geometry to help the model remain realistic: the niche should hold fewer than 400 cells total, row size should monotonically increase along the niche axis, and the geometry should be “well-behaved” (i.e., there should not be large jumps in row size along the axis).

#### Experimental set-up

We performed several sets of experiments aimed at discovering the effects that realistic niche geometry constraints have on the time to 300 cells. We therefore define a single statistic *y* to be the time to *N*=300 cells for a niche of *D* rows; a niche geometry vector ***θ*** defines the simulator input parameters. In this study we set the number of rows in the niche to *D*=8. To enforce the monotonicity constraints, we define *θ*_1_=1+*g*_1_ and *θ*_*d*_=*θ*_*d*−1_+*g*_*d*_,∀*d*>1, i.e. we define niche geometries in terms of niche increment parameters *g*_*d*_≥0. With this set-up, we can change the prior constraints and observe the effects on the posterior predictive distribution *p*(*y*|*y* ^⋆^).

There are three sets of constraints on ***θ*** (and/or **g**), each with their own kernel epsilon parameter; the constraint *g*_*d*_≥0 is strictly enforced. For all experiments, the first cell row was given a flexible range *θ*_1_∈{1,400}, thus the first constraint is $K_{\boldsymbol {\epsilon }_{g_{1}}}\left (g_{1};\; \tau _{g_{1}} \right)$, where $\boldsymbol {\epsilon }_{g_{1}}=0.1$ and $\tau _{g_{1}} = 399$. The second set of constraints is on the niche geometry increments $K_{\boldsymbol {\epsilon }_{g_{d}}}\left (g_{d};\; \tau _{g_{d}} \right) $, where $\boldsymbol {\epsilon }_{g_{d}} = 0.1$ and $\tau _{g_{d}}$ is set to 10 (to capture well-behaved niche increments) or 399 (essentially removing the constraint on niche increments); further experimental details are given below. The final constraint on ***θ*** is on the total niche geometry size $K_{\boldsymbol {\epsilon }_{\theta }}\left (\sum _{d=1}^{D} \theta _{d};\; \tau _{\theta } \right)$, where ***ε***_*θ*_=1 and *τ*_*θ*_ is set to 400 or 1500. For all experiments, a one-sided Gaussian kernel was used. The prior over **g** is therefore:

$$\begin{array}{*{20}l}  \pi\left(\textbf{g} \right) &\propto K_{\boldsymbol{\epsilon}_{\theta}}\left(\sum_{d=1}^{D} \theta_{d};\; \tau_{\theta} \right) K_{\boldsymbol{\epsilon}_{g_{1}}}\left(g_{1};\; \tau_{g_{1}} \right) \prod_{d=2}^{D} K_{\boldsymbol{\epsilon}_{g_{d}}}\left(g_{d};\; \tau_{g_{d}} \right) \end{array} $$

The likelihood is a one-sided kernel $\pi \left (y^{\star }\,|\, y\right) \propto K_{\boldsymbol {\epsilon }_{y}}\left (y;\; y^{\star } \right)$, where ***ε***_*y*_=0.01 (except for experiment D and E, below) and *y* ^⋆^=27.05. For this problem we did not know *y* ^⋆^ a priori, so we ran 5 runs of marginal kernel ABC with *S*=1 and adapted **y** ^⋆^ (Algorithm 2). We set *y* ^⋆^=27.05, the median value from 5 runs (which produced values 26.99, 27.03, 27.05, 27.07, 27.28). The EMA approach to estimating *y* ^⋆^ was fairly robust for this problem: since the EMA produces a local average of *y*, any improvement upon *y* ^⋆^ must be consistently better. The parameter ***ε***_*y*_ could be interpreted as an error in the estimation of *y* ^⋆^.

Table 1 summarizes the parameters and results from these experiments. For all experiments, 5 runs of marginal ABC-MCMC of length 10000 were conducted and the first 2000 samples were discarded as burnin.

**Table 1:** Stem-cell niche geometry experimental set-up and posterior predictive results

Experiment	M	*y* ^⋆^	$\tau _{g_{1}}$	$\tau _{g_{d}}$	*τ*_*θ*_	Mean *y*	Median *y*	Mode *y*	*P*(*y*<27.05)
A	1	27.05	399	399	400	27.042	27.037	27.029	0.53
	1	27.05	399	10	400	27.059	27.054	27.076	0.49
B	1	*∞*	399	399	400	27.078	27.081	27.076	0.43
	1	*∞*	399	10	400	27.298	27.150	27.114	0.32
C	1	*∞*	399	399	1500	30.159	30.184	30.224	0.00
	1	27.05	399	399	1500	27.322	27.227	27.150	0.24
D	10	27.05	399	399	400	27.053	27.049	27.043	0.51
	10	27.05	399	10	400	27.056	27.053	27.050	0.47

M is the number of replicates used to compute a statistics (see Experiment D). See text for definitions of other columns

#### Experiment A: realistic constraints on *g*_*d*_

**Fig. 1:**
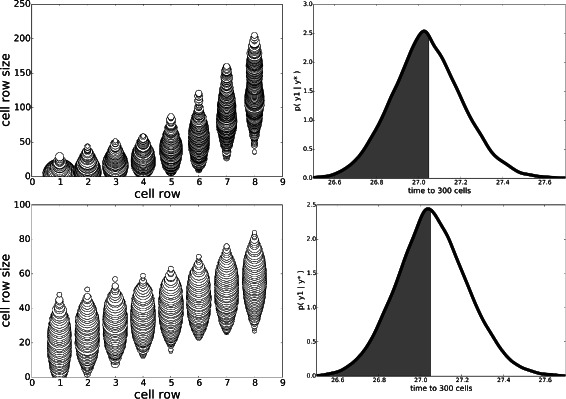
Stem-cell niche geometries, Experiment A. Comparison of niche geometry posteriors with $\tau _{g_{d}} = 400$ (top row) and $\tau _{g_{d}} = 10$ (*bottom row*). The *left column* illustrates the posterior geometries ***θ*** by plotting circles of radius proportional to their posterior fraction of that size for that row (rounded to integers). The *right column* is the posterior predictive distribution *p*(*y*|*y* ^⋆^), with shading indicating the probability mass *P*(*y*<27.05 | *y* ^⋆^)

The first set of experiments compared posterior inference using $\tau _{g_{d}}=399$ and $\tau _{g_{d}}=10$. Figure 1 shows the posterior geometries with $\tau _{g_{d}}=399$ (top row) and with a realistic constraint $\tau _{g_{d}}=10$ (bottom). Without the realistic constraint, the sizes start smaller (averaging around 5), increase slowly until row 6, then jump to a larger size (over 100) at row 8. With the realistic constraint, the sizes start larger (averaging around 20), and increase steadily until row 8, with no jumps, to an average of about 50. The posterior predictive distributions (Fig. 1, right) are very similar for both results, with the probability of *y*<27.05 without the constraint being 0.53 compared to 0.49 with the constraint, indicating that the constraints do remove some regions of the parameter space with shorter time to 300 cells. The medians and modes of *y*|*y* ^⋆^ also support this (without: 27.037/27.029, with: 27.054/27.076).

#### Experiment B: removing constraint on time to 300 cells

**Fig. 2:**
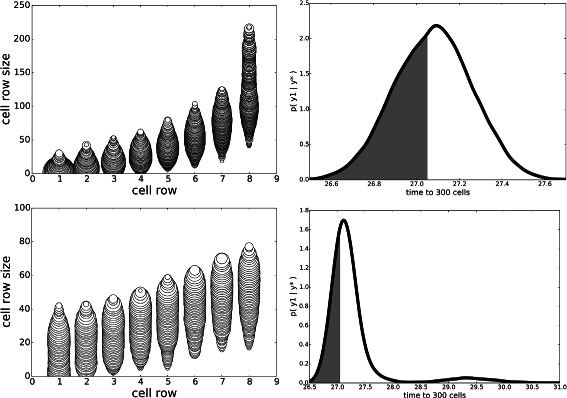
Stem-cell niche geometries, Experiment B. Comparison of niche geometry posteriors with $\tau _{g_{d}} = 400$ (*top row*) and $\tau _{g_{d}} = 10$ (*bottom row*), but with the likelihood term removed

We next removed the effect of the likelihood term on the posterior by setting *y* ^⋆^=*∞* (which is equivalent to sampling from the prior, with soft boundaries, using MCMC). Results for this experiment are shown in Fig. 2. Surprisingly, the posteriors of ***θ*** have the same form as in experiment A, though with some decreases in *P*(*y*<27.05 | *y* ^⋆^): from 0.53 to 0.43 (for $\tau _{g_{d}}=399$) and from 0.49 to 0.32 (for $\tau _{g_{d}}=10$). This result clearly shows that there is significant *prior mass* having *y*<27.05. However, it is unclear from this experiment what influence the other input constraints have on *y*, the time to 300 cells.

#### Experiment C: increasing threshold on total niche cells

**Fig. 3:**
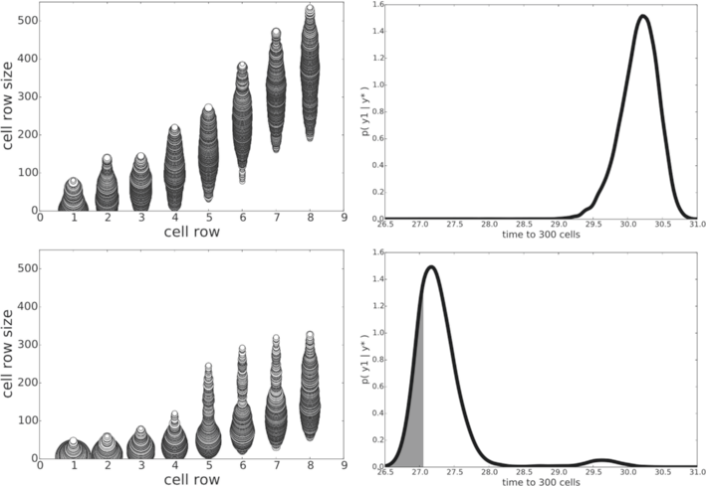
Stem-cell niche geometries, Experiment C. In these experiments, *τ*_*θ*_=1500. *Top*: *y* ^⋆^=*∞*. *Bottom*: *y* ^⋆^=27.05. When *y* ^⋆^=27.05, posteriors are similar to experiment A (Fig. 1, top)

In this experiment we increase *τ*_*θ*_=1500 in an attempt to determine the most important factor for minimizing the time to 300 cells: the likelihood constraint *y* ^⋆^ or the constraint on the total size. Results are shown in Fig. 3. By increasing the total niche geometry permitted and removing the constraint on *y* (Fig. 3, top row), the posterior predictive distribution degrades severely, with no samples satisfying *y*<27.05. However, when the constraint on *y* is reintroduced (Fig. 3, bottom row), a significant value of *P*(*y*<27.05 | *y* ^⋆^)=0.24 results; its posteriors of ***θ*** are also very similar to that of experiment A with $\tau _{g_{d}}=399$.

#### Experiment D: replacing statistics with average of replicates

**Fig. 4:**
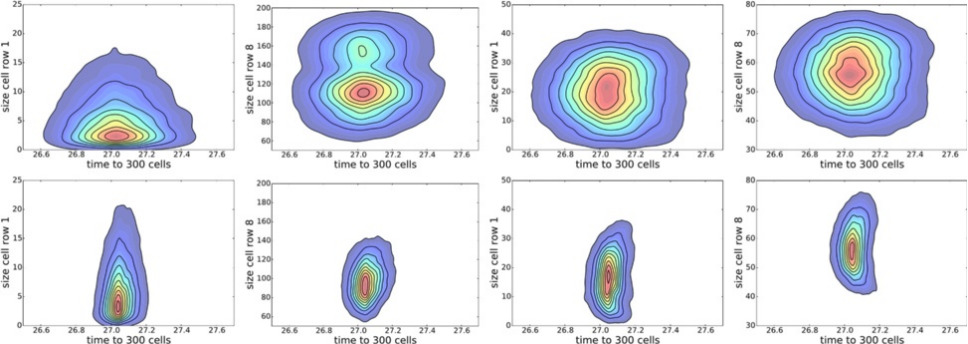
Stem-cell niche geometries, Experiment D. Effect of *M*, the number of replicates used to compute the output statistic *y*: *M*=1 (*top*) versus *M*=10 (*bottom*). The left 2 columns correspond to $\tau _{g_{d}}=399$ and the right 2 columns $\tau _{g_{d}}=10$. Each plot is a joint posterior *p*(*y*,*θ*_*d*_ | *y* ^⋆^), for *d*∈{1,8}

**Fig. 5:**
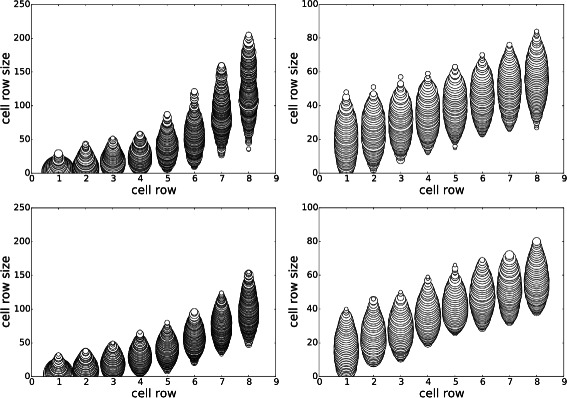
Stem-cell niche geometries, Experiment D. Effect of *M*, the number of replicates used to compute the output statistic *y*: *M*=1 (*top*) versus *M*=10 (*bottom*). The *left column* correspond to $\tau _{g_{d}}=399$ and the *right column* $\tau _{g_{d}}=10$

The aim of this experiment is to explore the effect that reducing the simulator noise has on the posterior distributions. To do this, we repeat each simulation *M* times, using the same parameter setting; i.e. $y = \frac {1}{M} \sum _{m=1}^{M} y^{(m)}$, where $y^{(m)} {\overset {\text {sim}}\sim } \pi (y | \boldsymbol {\theta })$. The variance of the statistic therefore decreases with *M*. Although, as expected, the posterior predictive distribution contracts around *y* (Fig. 4), we found no significant changes to the posterior *p*(***θ***|**y** ^⋆^) when *M*=1 (see Fig. 5). This experiment gives evidence that the scientist should instead change ***ε***_*y*_ to control the posterior predictive distribution rather than *M*, which has an *M*-fold increase in computation.

#### Experiment E: sensitivity to ***ε***_*y*_

**Fig. 6:**
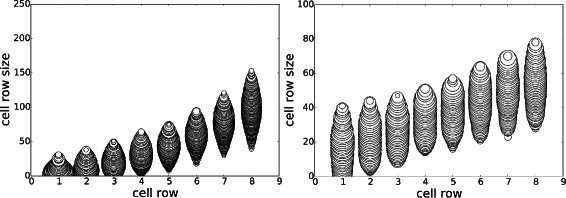
Stem-cell niche geometries, Experiment E. Comparison of niche geometry posteriors with $\tau _{g_{d}} = 400$ (left) and $\tau _{g_{d}} = 10$ (*right*) for ***ε***_*y*_=0.5. Compare with Fig. 1 where ***ε***_*y*_=0.01. See text for discussion

In this experiment we repeated experiment A but changed ***ε***_*y*_ from 0.01 to 0.5. This is a significant change if one considers the range of *y* in the posterior predictive distributions of the previous experiments. Results are shown in Fig. 6. For $\tau _{g_{d}} = 399$, the effect seems to be larger niche sizes for earlier rows when ***ε***_*y*_=0.5, resulting in final sizes smaller than when ***ε***_*y*_=0.01. For $\tau _{g_{d}} = 10$, there is a small effect on the niche geometry sizes; the distributions by row tend to be more uniform for ***ε***_*y*_=0.5 than for ***ε***_*y*_=0.01. The posterior predictive distributions for $\tau _{g_{d}} = 399$ worsened: mean *y* from 27.042 to 27.08 and *P*(*y*<*y* ^⋆^) from 0.53 to 0.43. A similar change occurred for $\tau _{g_{d}} = 399$: mean *y* from 27.059 to 27.12 and *P*(*y*<*y* ^⋆^) from 0.49 to 0.38. For small changes to ***ε***_*y*_, we found very little change in the posterior (not shown). This confirms our results that the constraints **y** ^⋆^ are the main influence on the posterior. It is only by making ***ε***_*y*_ relatively large that the results become significantly different. In fact, this difference is similar to that observed between Experiments A and B, where the entire constraint $y_{1}^{\star }$ is removed.

#### Discussion

The results of experiments A-C demonstrate the relative importance of the input and output constraints on the posterior probability of *y* | *y* ^⋆^. The most important constraints are $\sum \theta _{d}$ and *y*<*y* ^⋆^. Both have similar effects on the posterior predictive distribution. The constraint $\tau _{g_{d}}$ has little effect on *P*(*y*<27.05 | *y* ^⋆^), but does produce significantly different posterior geometries, mainly due to the prior constraints.

Experiments A-D illustrate the usefulness of POPE for exploring the roles constraints play on the optimization posterior. We found that the constraints on the prior over valid regions of ***θ*** had significant influence on the posterior, and played a role similar to the likelihood term. Using realistic constraints on changes in row sizes had very little detrimental effect on the time to 300 cells, compared to having no realistic constraint. More important was the constraint on total geometry size. We found very little difference in the posteriors when the statistics were averages of simulation replicates versus a single simulation. This makes sense if the simulation noise is taken into account when setting ***ε***: when increasing the number of replicates in the average, ***ε*** should be decreased (from its setting at *M*=1) to take into account the population mean variance, but this seems unnecessary since the posteriors change little, but the number of simulations increases.

Experiment E explored the role ***ε***_*y*_ plays in POPE. We found that only large changes had significant effects on the posterior, though we emphasize that this is entirely problem specific. In the niche experiments, there is a large region of parameter space which satisfies all the constraints. For this problem, ***ε*** plays a less important role than in other problems where it is difficult to find any parameter values for which all constraints are satisfied. In those situations, ***ε*** plays a critical role in POPE since it enables mixing of the Markov chain. It is therefore useful to measure acceptance probabilities in a few preliminary runs to guide the scientist in setting ***ε***. Once a satisfactory acceptance rate is achieved (e.g. 20 *%* to 40 *%*), one could fix ***ε*** and run experiments. Afterwards, samples that violate constraints can always be ignored in the analysis.

From a biological perspective, further simulation experiments with this stem-cell model could address whether giving cells some flexibility in the position at which they differentiate allows for more flexibility in the optimal geometry, perhaps allowing that geometry to also satisfy competing performance objectives. POPE offers a robust and consistent Bayesian framework for new experiments.

### Case 2: spotted patters in colon cancer tumors

A remarkable pattern of spots is visible in the tissue of colon cancer tumors when stained for markers indicating glycolytic activity. It is hypothesized that the spotted regions indicate localized areas of glycolytic cells, whereas surrounding areas are considered oxidative cells. Furthermore it is thought that Wnt signaling (an important cell signaling pathway in development and healing) plays a critical role in reducing glycolytic activity [26], thereby resulting in significant changes in spot formation. Experiments blocking Wnt by overexpression of a dominant negative form of lymphoid enhance factor (dnLEF-1) have shown that interfering with the Wnt pathway leads to fewer but larger spots and lighter background staining color than *Mock* tissue (tumors that have not received dnLEF-1 intervention).

Based on these findings, a simulator of a mathematical model of reaction-diffusion equations was built that produces spatial and temporal dynamics of a population fraction of oxidative cells and glycolytic cells, as well as the activity of Wnt and a Wnt inhibitor. The Wnt and Wnt inhibitor equations are based on the Gierer-Meinhardt activator-inhibitor model, where Wnt is the activator which produces a factor that inhibits Wnt activity.

**Fig. 7:**
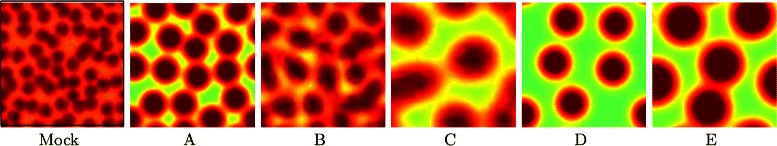
2D simulation patterns of glycolic cells at the final time step. See text for details

The goal of these experiments is to provide feedback to the mathematical biologists regarding the characteristics of simulation parameters that produce *simulated patterns different from Mock patterns*. For this reason, this problem does not have a predefined cost function, but instead uses the observed Mock values as constraints. The simulation produces 1D spatial and temporal patterns (see Fig. 7 for 2D examples) from which *J*=4 statistics are computed: *y*_1_ the average spot width (based on wave patterns in 1D images); *y*_2_ the number of spots (waves, in 1D); *y*_3_ the average background level; and *y*_4_ the average Wnt level. There are *D*=9 simulator parameters including rates of production and decay for Wnt and Wnt inhibitor, and their diffusion coefficients. These are described in Table 2. The ***θ*** settings in column *Mock* in Table 2 generate patterns that were judged similar to the Mock spotting patterns in tissue photographs. Their corresponding statistics **y** ^⋆^={0.604,5,0.807,5.67} are shown in Table 3, along with statistics from other ***θ*** settings *A* to *E*, described below.

**Table 2:** Simulation parameters *θ* for spotted patterns in colon cancer tumors

Parameter *θ*	Description	Mock	A	B	C	D	E
*κ*_*W*_>0	Rate of nonlinear Wnt production	4	0.442	0.951	2.44	0.399	0.315
$\kappa _{W_{I}} > 0$	Rate of Wnt inhibitor production	1	27.4	0.484	0.161	0.486	0.188
*μ*_*W*_≥0	Decay rate of Wnt	2	0.642	0.179	0.791	0.545	0.936
$\mu _{W_{I}} \geq 0$	Decay rate of Wnt inhibitor	4	2.36	1.30	1.10	0.569	1.064
*a*≥0	Constant of inhibition	10^−8^	0.4006	0.416	0.0384	0.00491	0.0284
*b*≥0	Constant of inhibition by *W*_*I*_	1	0.0125	7.94	20.05	0.616	0.640
*S*_*W*_≥0	Rate of constitutive Wnt production	1	0.00167	0.00351	17.75	0.00005	0.00009
1≥*D*_*W*_>0	Diffusion coefficient of Wnt	0.01	0.0180	0.00322	0.0955	0.0336	0.0810
1≥*N*>0	Nutrient level	1	0.818	0.897	0.984	0.959	0.970

**Table 3:** Simulation statistics *y* for spotted patterns in colon cancer tumors

Statistic *y*	Feasible region	Mock (*y* ^⋆^)	A	B	C	D	E
Avg. Spot Width	*y*_1_>0.604	0.604	1	0.65	0.65	1	1.75
Number of Spots	*y*_2_∈[2,3,4]	5	3	4	2	3	2
Avg. Background	*y*_3_<0.807	0.807	0.77	0.75	0.70	0.6	0.70
Avg. Wnt	*y*_4_<5.67	5.67	3.25	1.50	0.75	1	2

The Mock values **y** ^⋆^ define the constraints on simulator statistics **y**. More precisely, they constrain the posterior to regions where $\left [ y_{1} > y^{\star }_{1}\right ],\left [ y_{2} < y^{\star }_{2}\right ],\left [ y_{3} < y^{\star }_{3} \right ]$, and $\left [ y_{4} < y^{\star }_{4} \right ]$, which correspond to the goal of producing different patterns from Mock. For example, the first constraint states that we want the spot widths from simulation to be greater than $y^{\star }_{1} = 0.604$, the average width of spots for the Mock setting ***θ***. Similarly, we want fewer than 5 spots, a background lighter than 0.807, and a Wnt level less than 5.67. Further constraints are added to avoid degenerate simulation results; as an example, we set its likelihood to zero when there are no spots detected.

This simulator is deterministic but expensive to evaluate, requiring roughly 30 seconds to complete for the 1D simulator used in our experiments, and 90 seconds for the 2D simulator, used for generating 2D images only. We ran 6 chains of length 4000 pseudo-marginal kernel ABC-MCMC with S=1. To initialize the chains, a short rejection sampling procedure was used to select ***θ***_0_ for each random seed. This is necessary as many random configurations of ***θ*** result in degenerate simulation results (i.e. zero likelihood). Diffuse log-normal prior distributions were placed over *θ*_1_ to *θ*_7_ and weak Beta priors put on *D*_*W*_ and *N*. At least 100 initial samples were discarded from each chain; sometimes more if the chain had not yet reached a location where all the constraints were satisfied. In total there were 22257 samples in the posterior.

**Fig. 8:**
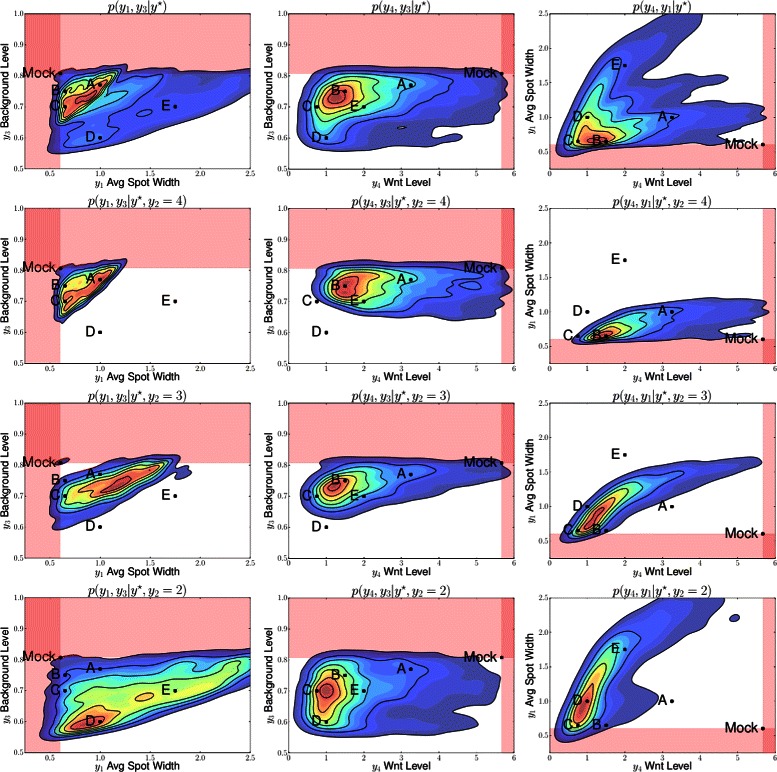
Posterior predictive distributions (PPDs) shown marginally for pairs of statistics. Row 1: The full PPD. Rows 2 to 4: spot-conditional PPDs for spot numbers 4 to 2, respectively. Columns differ on pairs of statistics. Mock constraints indicate invalid regions in shaded pink. Interesting posterior modes are labeled *A*-*E*

**Fig. 9:**
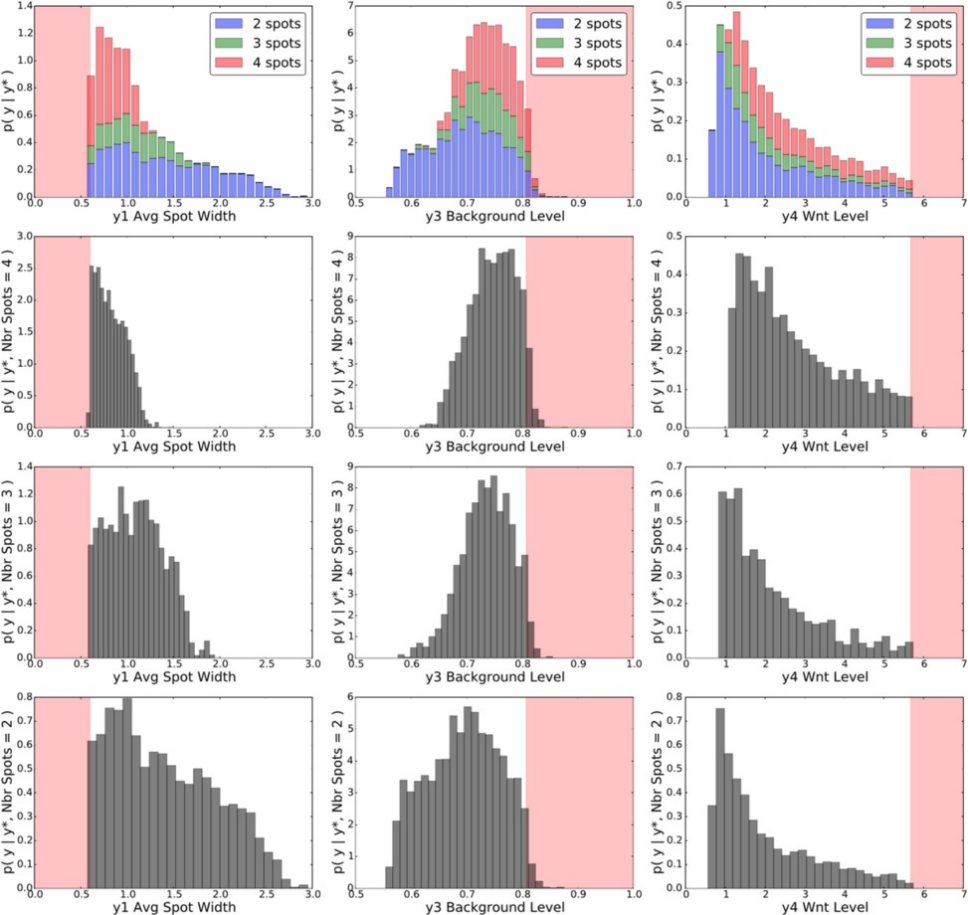
Marginal posterior predictive distributions (PPDs); one column for statistics *y*_1_,*y*_3_, and *y*_4_. Row 1: The full PPDs as histograms, but using colors *blue* (*y*_2_=4), *green* (*y*_2_=3), and *red* (*y*_2_=2) to differentiate spot numbers. Rows 2 to 4: spot-conditional PPDs for spot numbers 4 to 2, respectively

**Fig. 10:**
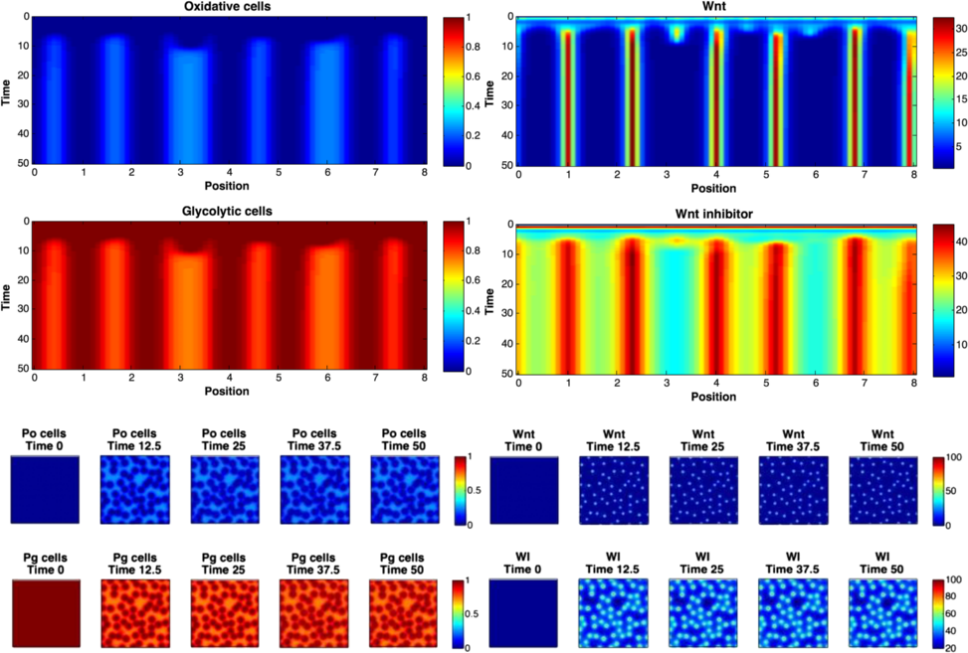
Simulator outputs for the Mock setting of *θ*. *Upper plots:* 1D simulation results. Images show the concentration of oxidative, glycolytic cells (left) and concentration of Wnt and Wnt inhibitor (right), spatially and temporally. *Lower plots:* 2D simulator results. Temporal slices of 2D spatial concentrations of oxidative (Po), glycolytic (Pg) cells (left) and Wnt and Wnt inhibitor (right)

**Fig. 11:**
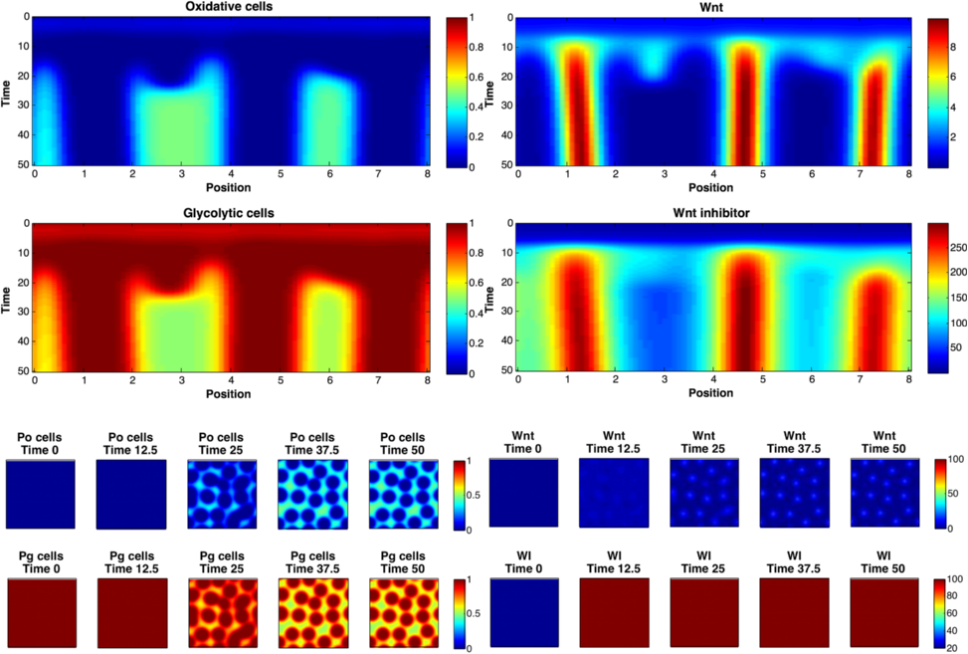
Simulator outputs for ***θ*** setting *A*

**Fig. 12:**
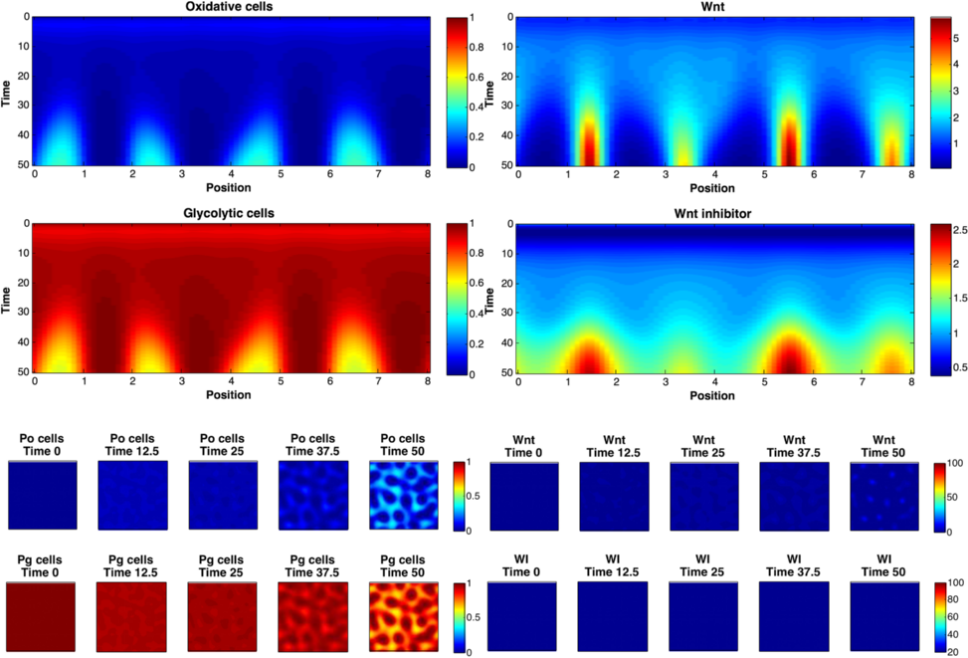
Simulator outputs for *θ* setting *B*

**Fig. 13:**
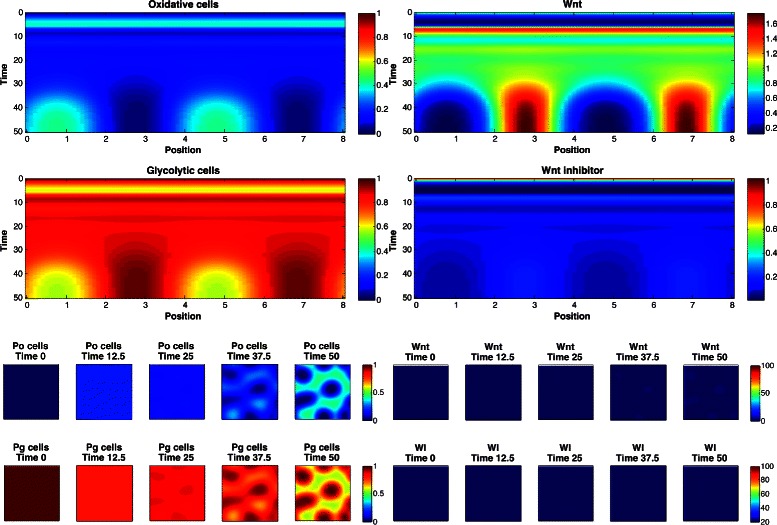
Simulator outputs for *θ* setting *C*

**Fig. 14:**
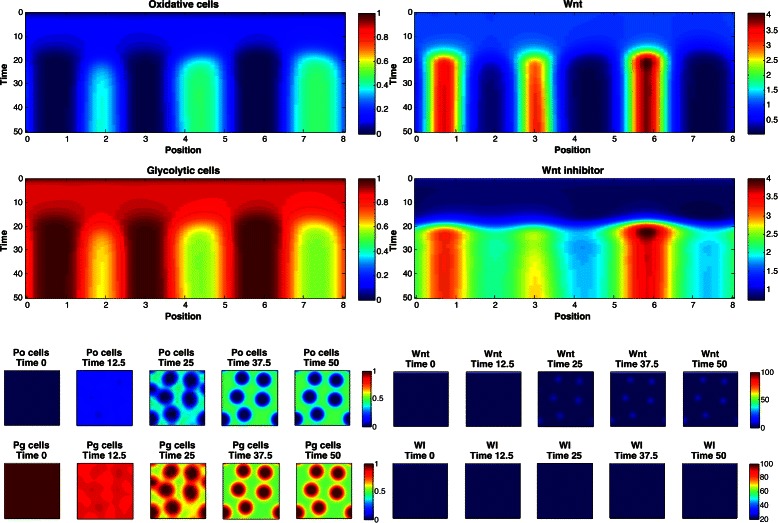
Simulator outputs for *θ* setting *D*

**Fig. 15:**
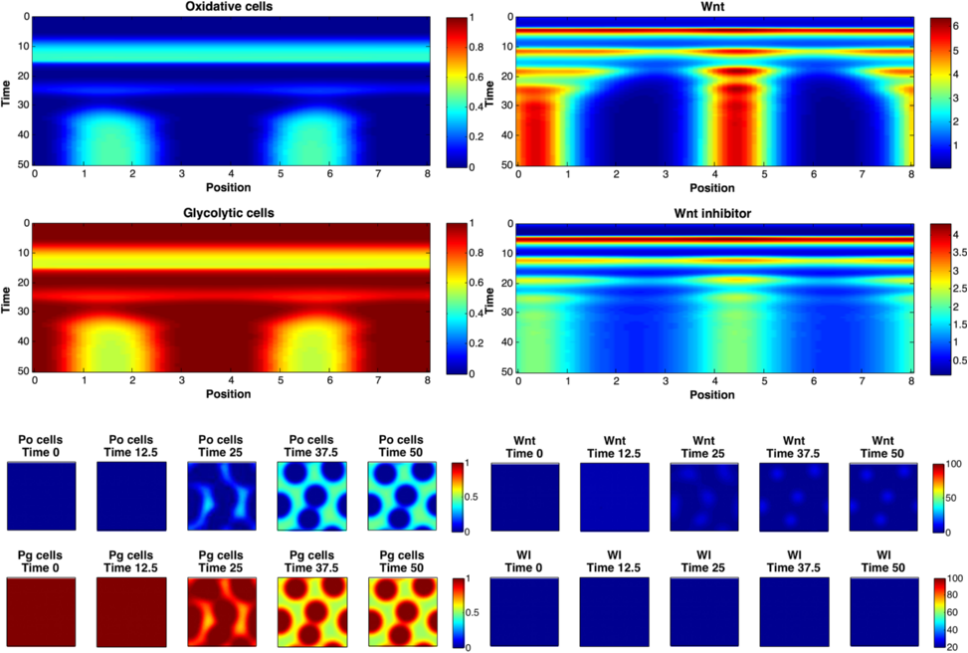
Simulator outputs for *θ* setting *E*

Analysis of the posterior predictive distribution revealed distinct distributions when conditioned on *y*_2_, the number of spots. The posterior distribution can therefore be viewed as a mixture of 3 spotting patterns, with *p*(*y*_2_ | **y** ^⋆^)=[0.505,0.185,0.310], where *y*_2_∈{2,3,4}. The marginal posterior predictive distributions are shown in Fig. 8 for pairs of statistics, and in Fig. 9 for marginal distributions. To illustrate the role of the spotting patterns, by visual inspection of the posterior predictive distributions displayed in Fig. 8, we selected statistics labeled *A* through *E*. Parameters ***θ*** corresponding to the modes *A*-*E* were ran in both the 1D and 2D simulator producing images in Fig. 7, showing the desired shift away from Mock patterns. Figures 10, 11, 12, 13, 14 and 15 provide full illustrations of the 1D and 2D simulations of Mock and patterns *A*-*E*.

**Fig. 16:**
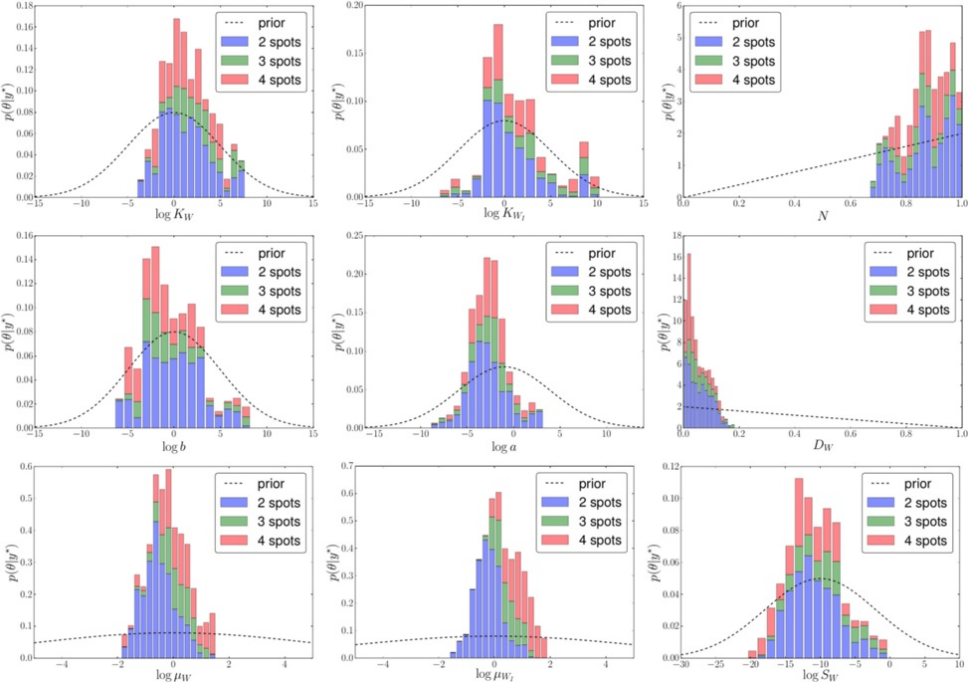
Marginal posterior parameter distributions.Each figure shows the histogram for the marginal posterior distribution using colors *blue* (*y*_2_=4), *green* (*y*_2_=3), and *red* (*y*_2_=2) to differentiate spot numbers (associated with the simulator statistics run at their parameter setting). The prior *p*(*θ*) is also shown as a dashed line. Parameters *μ*_*w*_ and *μ*_*WI*_ have the most distinct spot-conditional distributions

**Fig. 17:**
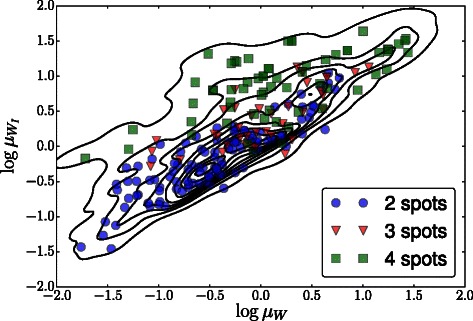
The posterior distribution of log*μ*_*W*_ versus $\log \mu _{W_{I}}$. Overlaid are subsamples from the posterior with colored symbols indicating the number of spots its setting produced, showing the strong relationship between these parameters and the number of spots

Spot distributions were also found for *p*(***θ*** | **y** ^⋆^), most distinctly for the Wnt and Wnt inhibitor decay rates (*μ*_*W*_ and $\mu _{W_{I}}$, respectively), which showed decreasing value for fewer spots, validating the original experimental hypothesis that blocking Wnt production by dnLEF-1 overexpression leads to qualitatively different spotting patterns. The marginal posteriors are shown in Fig. 16, along with the prior, for reference. The strong relationship between *μ*_*W*_ and $\mu _{W_{I}}$ is shown in Fig. 17. Subsamples from the posterior are overlaid with markers indicating the number of spots.

**Fig. 18:**
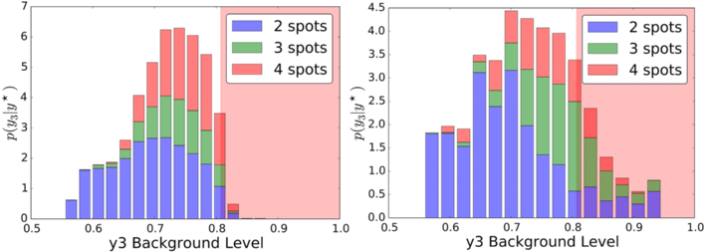
Effect of increasing *ε* on posterior predictive distribution *p*(*y*_3_|*y* ^⋆^). *Left:* *ε*={0.01,0.05,0.01,0.05} (repeated from Fig. 9). *Right:* *ε*={0.1,0.5,0.1,0.5}. See text for details

Similar to Experiment E for Case 1, we examined the effect increasing ***ε*** has on the posterior (predictive) distributions. The main difference for this case is that since **y** ^⋆^ defines a set of constraints on the simulator outputs, the effect of increasing ***ε*** is to tolerate larger constraint violations as measured by the one-sided kernel likelihood evaluations. For this experiment we increased ***ε*** by an order of magnitude from ***ε***={0.01,0.05,0.01,0.05} to ***ε***={0.1,0.5,0.1,0.5}. Figure 18 shows one posterior predictive distribution for the two sets of ***ε***, clearly demonstrating the increased number of constraint violations in the posterior for larger ***ε***. For ***ε*** set too high, there is an increased amount of wasted computational effort. Although we want some slack in violating constraints, too much allows the Markov chains to wander far from the region of interest. As mentioned for the stem-cell niche case, setting the values of ***ε*** are problem specific. Because it is the constraints **y** ^⋆^ that contribute most significantly to the likelihood, small changes in ***ε*** have minor effects on the posterior and it is only when large changes are made that the differences become important.

#### Discussion

This case study illustrates the usefulness of POPE for exploratory simulation analysis. As a first attempt at studying this simulator from an ABC perspective, POPE revealed several regions of parameter settings that produce qualitatively different images from Mock. Now experts can examine these various solutions to further develop the simulator or to increase the number of statistics. For example, some of the parameter settings in the posterior seem to be similar to the prior, indicating they have little influence on the posterior. If this does not match the intuition of the experts, the role these parameters have in the simulator can be re-evaluated. The *J*=4 statistics may also not be the most informative for the experts; based on our results, learning the statistics (using computer vision techniques applied to the images) or modifying the current statistics may improve the ability of the experts to learn more about the spot formation process.

This type of interaction between simulation model and cancer researchers is important; ongoing research with a modified version of this tumor metabolism simulator will include non-constant nutrient levels and various therapeutic regimes, which will improve our understanding of cancer metabolism, and in turn aid the development of new treatments or therapies.

## Conclusions

In simulation-based science, simulators encode complex models of natural phenomena. Often scientists wish to find an optimal parameter setting—one that minimizes some cost function—for their simulator, subject to constraints on both the parameters and the other outputs of the simulator. However, a single optimal parameter setting, while useful, conveys limited information to scientists about parameter dependencies and sensitivities or allow them to compare different models in terms of their goodness of fit. We have proposed simple extensions to likelihood-free inference that incorporate one-sided likelihood kernels into standard ABC algorithms, allowing scientists to run ABC, post-optimization.

With POPE, scientists can answer these important questions regarding their optimized simulation model using a fully Bayesian approach. As a result, scientists can examine posterior predictive distributions, parameter correlations and perform sensitivity analyses. These analyses could in turn discover “overfit” optimum, where minor changes to the parameters lead to dramatic changes in the cost function, or quickly violate (biological) constraints. As Bayesian inference procedure, POPE naturally incorporates parameter and simulator uncertainty, therefore allowing it to be used to discover regions of parameter space that improve upon optimal settings.

We applied POPE to two case studies: one in an optimization setting (stem-cell niche geometry) and a non-optimization setting (spotting patterns in cancer tissue), showing its usefulness to *general* constraint-based likelihoods. These studies demonstrated that POPE naturally handles constraints on both the input parameters and the simulator output statistics, as well as in situations where the simulator is either very noisy or is deterministic. The preliminary results on these case studies offer many avenues for future work.

It is natural to extend POPE with surrogate models so that it can be applied to expensive simulators. Although there is considerable excitement in the machine learning community about optimizing objectives that are hard to evaluate, such as those defined by simulators, there is almost no work on analyzing such problems “post optimization”. POPE is easily combined with black-box optimization using surrogates with Bayesian posterior inference.

## Endnotes

^1^ Note that this is reminiscent of the “probability of improvement” used in Bayesian optimization [8]. However, that quantity is different due to the fact that it includes the uncertainty of the surrogate function to predict the value of **y**. In POPE the posterior probability density is solely determined by the uncertainty due to noise in the simulation process.

^2^ We wish to clearly distinguish between response models described here and a simulator as a model of natural phenomena. A response model is a conditional distribution of statistics **y** at parameter location ***θ*** and has its own sets of parameters, such as mean and variance, or Gaussian process covariance parameters, that are of secondary interest and are useful computationally for inference. These should be distinguished from simulator parameters ***θ*** that are scientifically interesting, in of themselves.
